# Supplementary material for: Building regulatory landscapes reveals that an enhancer can recruit cohesin to create contact domains, engage CTCF sites and activate distant genes
Source: Nat Struct Mol Biol. 2022 Jun 16;29(6):563–74. doi: 10.1038/s41594-022-00787-7 (PMC9205769; doi:10.1038/s41594-022-00787-7)
Supplement: Supplementary file 1 — Supplementary Tables 1 and 2 and Supplementary Figure 1. [file 41594_2022_787_MOESM1_ESM.pdf]

nature portfolio

https://doi.org/10.1038/s41594-022-00787-7

Supplementary information

Building regulatory landscapes reveals that an enhancer can recruit cohesin to create contact domains, engage CTCF sites and activate distant genes

In the format provided by the authors and unedited

Table S1. PCR Primers Sequences

Homology arms targeting constructs

pos-50_Left_HA_FWD	AACACGCTAGGTGTACTGCC
pos-50_Left_HA_REV	ACGCACAGCTTTGACAAAAGG
pos-50_Right_HA_FWD	GGGGCCCCAGCCCTAGT
pos-50_Right_HA_REV	TGAAAGTACCATGGGCGTTGT
pos0_Left_HA_FWD	GTGTGTGTGCTGGCTGATGAC
pos0_Left_HA_REV	TGCCCGAAGGACCGCGC
pos0_Right_HA_FWD	ACGTCGGCGGTGACGGTGAAG
pos0_Right_HA_REV	GTCACCGAGCTGCAAGAACTC
posMin0_Left_HA_FWD	GCATGGATGTCCAGTGATACA
posMin0_Left_HA_REV	TACACCTCCAATTGACTCAAATG
posMin0_Right_HA_FWD	AGGTGTACCTGTGGATGTATTT
posMin0_Right_HA_REV	AGACCACAGTTTCAGCGCA
pos11_Left_HA_FWD	CATGAGAGCCCACCCCTATTC
pos11_Left_HA_REV	AGGAGGGGACAGAAAGCAAATC
pos11_Right_HA_FWD	CCCCTTTGGTTCAGAATGTCA
pos11_Right_HA_REV	TGTACGGACTTCATGCCAGG
pos47_Left_HA_FWD	TAGGTCACTCCCTCCATCCTC
pos47_Left_HA_REV	CAGGAGCTGACTGGGGACT
pos47_Right_HA_FWD	AAGTAGCTACCAACCCAGGC
pos47_Right_HA_REV	AACAAAACTTATGAGGTCCATCAAT
pos100_Left_HA_FWD	CCAACATTCCCAGAACCAAAC
pos100_Left_HA_REV	CTGAAAACGGTGAGGTTTCTG
pos100_Right_HA_FWD	GGAGGTTAAATTTTGCCCACT
pos100_Right_HA_REV	GGTCCCTAGAGTTCTGTGTCAC
pos407_Left_HA_FWD	TTTTCCACCTGCATCTGCCT
pos407_Left_HA_REV	ACCCTGTTGTCTACCTAGAGAGG
pos407_Right_HA_FWD	TGGGGAAACACTGAGTAATGGT
pos407_Right_HA_REV	CCATCCCATGGGCTTGCTAT
dsRed_Left_HA_FWD	AGCACTCACATCTTGCCACT
dsRed_Left_HA_REV	AGGGGCCCACTGAAGAG
dsRed_Right_HA_FWD	AGGGGAGGAAGTGAGAGACA
dsRed_Right_HA_REV	GTTCAGCATGGACTAGGGGG

human 3x CTCF

CTCF_STAM_A2_FWD	AGAGCGAGATTCCGTCTCAA
CTCF_STAM_A2_REV	AGGACAAGCAACAATGGCTGGCCCATAGTA
CTCF_STAM_A4_FWD	TGGGCCAGCCATTGTTGCTTGTCCTTCCTTCCTGT
CTCF_STAM_A4_REV	CCTGCAAACTGAACTCCTGACCCCTCACAA
CTCF_STAM_E2_FWD	TCAGGAGTTCAGTTTGCAGGTGGCTTGACT
CTCF_STAM_E2_REV	TTTGATTTCCTTCACTCTGGAA

Genotyping primers

uLCR_FWD	CAAGGTTTCACTCTGCTGTC
uLCR_REV	TGAATGAGGCTTGAGTACAG
3xhCTCF_FWD	TCTAGATTAGACATAGGCAAGCACA
3xhCTCF_REV	TCTGCCACTGCCTAGTTGAG
3xmCTCF_FWD	TGTCCAACAGAAGATCTTACCACA
3xmCTCF_REV	CTAGTGTGGGCATCGTCCAG

dsRed_FWD	CCCCTAAGCTATCAGGTTGATTGA
dsRed_REV	AGCAATAGCATCACAAATTTCACA
pos-50_Left_HA_FWD	GCATTATTCATAATAGCACATCTCAATTC
pos-50_Right_HA_REV	TGGCACAGCTATATCTAAGGCG
pos-50_noInsert_FWD	GCATTATTCATAATAGCACATCTCAATTC
pos-50_noInsert_REV	TGGCACAGCTATATCTAAGGCG
posMin0_Left_HA_FWD	CAGGACAGGGGATGAGCTT
posMin0_noInsert_FWD	TCCGAGAATTCCAGAAAATGATG
posMin0_noInsert_REV	ACCTCCCCCTGAACCTGAA
pos0_Left_HA_FWD	GTATTTACCACTGGATAAGTG
pos0_Right_HA_REV	AGCTTACCATGACCGAGTAC
Pos0_noInsert_FWD	TCCTGTGTAAAGCTGGATCC
Pos0_noInsert_REV	ACTTCAACTGTAGGCGTCTC
pos11_Left_HA_FWD	GCTCTGGCTCCGTAGAAGTTG
pos11_Right_HA_REV	TGGGCACGTGATGGGAGATA
Pos11_noInsert_FWD	CTCAACATGCCCCTCTCCTG
Pos11_noInsert_REV	TTTGAAGCTGAGGAGCGACA
pos47_Left_HA_FWD	TTGGAATTCTCAGTTCCATCACA
pos47_Right_HA_REV	ACTGAGGGGAGGCTTTTAACT
Pos47_noInsert_FWD	TCCCCCAATTCTGCAGGTTC
Pos47_noInsert_REV	GGCCAGTCTCAGTGGTTCAT
pos100_Left_HA_FWD	CTAAGAGAAACAAACGCCAAC
pos100_Right_HA_REV	AAGATGAATTGAAAGGAGGTC
Pos100_noInsert_FWD	CAGCATCACTGGCCTAACCT
Pos100_noInsert_REV	CTCTTCTGCCACCAGGCTAT
pos407_Left_HA_FWD	TCATACCAACAGGATCACAAAACA
pos407_Right_HA_REV	TGACTGATTCAACAGGGTGCTTT
Pos407_noInsert_FWD	TCATACCAACAGGATCACAAAACA
Pos407_noInsert_REV	TGACTGATTCAACAGGGTGCTTT
posdsRed_Left_HA_FWD	TGGCTGAAATAGGGCAGCAT
posdsRed_Right_HA_REV	CGTCACTTACATTTTCCACTGC
small_domain_FWD	CCCCTAAGCTATCAGGTTGATTGA
small_domain_REV	TCCTTACTGTAGCCTGTGGA

ChIP-qPCR

SMC1_pos_control_FWD	CTGAAGATCCCCTGTGCGACC
SMC1_pos_control_REV	ACTACCCAAGGGGATCGAAGC
H3K27me3_pos_control_FWD	TCCAGATGTGCAGTCGTGTT
H3K27me3_pos_control_FWD	TGGCCACACTTTGGAGTTCA
Left_boundary_FWD	TGAAACTTGTGGACATGCCTTATC
Left_boundary_REV	AAGAACTGCATTGTGGGATGGAA
3xhCTCF_FWD	TTCAGTCCTTTAGCGCCACC
3xhCTCF_REV	GTCAAGCCACCTGCAAACTG
3xmCTCF_FWD	CTCTTCTGCTCCACCTGCAA
3xmCTCF_REV	AGCCTTAGAGAGGGGTCCAG
3'GFP_FWD	CTACCCTCCTCTGAGAAAACTCC
3'GFP_REV	CCTGTGCTTCTGCTAGGATCAA
HBG1_promoter_FWD	TTCAGGGTCAGCTTGCCGTA
HBG1_promoter_REV	ACACTCGCTTCTGGAACGTC

4C primers Ectopic viewpoints

GFP_FWD	GGGGCACAAGCTGGAGTA
GFP_REV	GCTCCTGGACGTAGCCTTC
uLCR_FWD	TTTAATATGCTTTAAGTTCTGGGGTAC
uLCR_REV	CTGACCCCGTATGTGAGCA
3xhCTCF_FWD	GGAATCTCGCTCTGATCGTC
3xhCTCF_REV	TGCAGGTGGCTTGACTG
3xmCTCF_FWD	ATTCTCTGCTAGTGTGGGCATC
3xmCTCF_REV	GACCCCTCTCTAAGGCTGACA

4C primers Endogenous viewpoints

Left_boundary_FWD	ATCAAAAATGAGTGAAAGGT
Left_boundary_REV	CTAGTTGCTTCGTGTGTTCA
Pos0_FWD	TTGGCGGCTGCAAGATAC
Pos0_REV	AGACCCCCAAACTCCGATT
Pos11_FWD	GGAAACCTCAGGACTAGGCAT
Pos11_REV	CCCTGATGGTATCGCTGAAT
Pos47_FWD	AAGGAGAACAAGGCCTTCAGTA
Pos47_REV	TCCTTCCAACCAGCACTCATAG
Pos100_FWD	ACAGGGTCTCACTCTGTGGAG
Pos100_REV	TTACATTTATATGTGCACAGCAAGTC
Pos407_FWD	GGGGTTTCCAGGCAAGTA
Pos407_REV	TTGCCATTTCCACCAAGGTC

Table S2. oligos for sgRNA cloning

For targeting regulatory elements

pos-50_upper	caccgTCAAAGCTGTGCGTCAAGTT
pos-50_lower	aaacAACTTGACGCACAGCTTTGAc
3'GFP_cut#1_upper	caccgTACCTGTGGATGTATTTCAA
3'GFP_cut#1_lower	aaacTTGAAATACATCCACAGGTAg
GFP_middle_upper	aaacCGGCGCGGGTCTTGTAGTTGc
GFP_middle_Lower	caccgAGCACTGCACGCCGTAGGTC
3'GFP_cut#2_upper	caccGTCAATTGGAGGTGTACCTG
3'GFP_cut#2_lower	aaacCAGGTACACCTCCAATTGAC
pos0_upper	caccgCACCGTCACCGCCGACGTCG
pos0_lower	aaacCGACGTCGGCGGTGACGGTGc
pos11_upper	caccgTTCTGAACCAAAGGGGTGCC
pos11_lower	aaacGGCACCCCTTTGGTTCAGAAc
pos47_upper	caccGGTAGCTACTTCTATAGAGC
pos47_lower	aaacGCTCTATAGAAGTAGCTACC
pos100_upper	caccGAAACCTCACCGTTTTCAGG
pos100_lower	aaacCCTGAAAACGGTGAGGTTTC
pos407_upper	caccGGTAGACAACAGGGTACTTG
pos407_lower	aaacCAAGTACCCTGTTGTCTACC
posdsRed_upper	caccgCCCCTGATTTGGTGCATGGC
posdsRed_lower	aaacGCCATGCACCAAATCAGGGGc
E407_downstream_upper	caccgATTCATTGGCCTCTCCGTTT
E407_downstream_lower	aaacAAACGGAGAGGCCAATGAATc
dsRed_upstream_upper	caccgTGACTTTGGGGGTCTAGAAT
dsRed_upstream_lower	aaacATTCTAGACCCCCAAAGTCAc
Right_boundary_upper	caccgAATTTTCACAAAATTCGGAC
Right_boundary_lower	aaacGTCCGAATTTTGTGAAAATTc

For KRAB silencing

sg_non-targeting_Control	ttgGTGCACCCGGCTAGGACCGGgtttaagagc
sg_non-targeting_Control	ttagctcttaaacCCGGTCCTAGCCGGGTGCACcaacaag
sg_SMC1A_upper	ttgGGCCGTACGCCCGAGAACTGgtttaagagc
sg_SMC1A_lower	ttagctcttaaacCAGTTCTCGGGCGTACGGCCcaacaag
sg_SMC3_upper	ttgGGGAGCGAGCGGCGCTTTGGgtttaagagc
sg_SMC3_lower	ttagctcttaaacCCAAAGCGCCGCTCGCTCCCcaacaag
sg_RAD21_upper	ttgGAAGGAGGCGCCGGCTGTGGgtttaagagc
sg_RAD21_lower	ttagctcttaaacCCACAGCCGGCGCCTCCTTCcaacaag
sg_MED21_upper	ttgGGTTTGCTGCGGTAGGAACAgtttaagagc
sg_MED21_lower	ttagctcttaaacTGTTCCTACCGCAGCAAACCcaacaag
sg_GATA1_upper	ttgGTGAGCTTGCCACATCCCCAgtttaagagc
sg_GATA1_lower	ttagctcttaaacTGGGGATGTGGCAAGCTCACcaacaag

Supplementary Information Figure 1

a

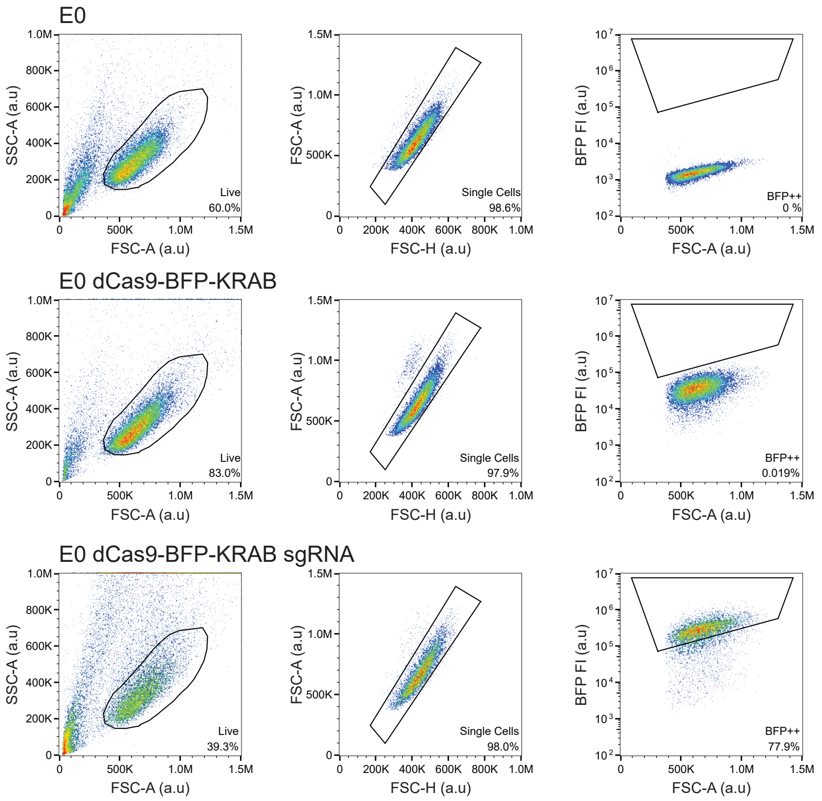

b

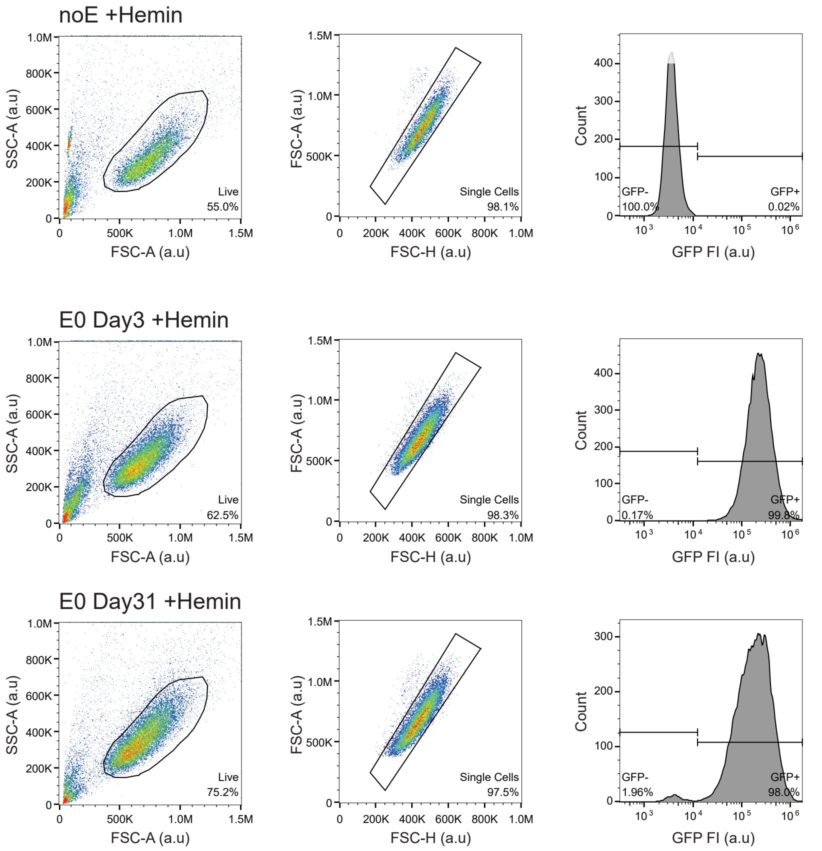

Supplementary Information Fig. 1 | Gating strategy for flow cytometry analysis

a. Gating strategy for GFP FI analysis of cells without (top) and with knockdown of a given factor (middle and bottom row). From left to right: Cells were gated for live (FSC-A and SSC-A), single cell (FSC-A and FSC-H) and for BFP. Top row shows E0, middle row E0 dCas9::KRAB and lower row shows E0 dCas9-BFP-KRAB transduced with a lentiviral vector carrying the sgRNA and a BFP expression cassette. Note that dCas9-KRAB gives intermedium BFP expression levels, while the introduction of the sgRNA-BFP vector yields high BFP expression levels (BFP++); BFP++ gating was based on E0 dCas9::KRAB.

b. Gating strategy for silencing experiments. From left to right: Cells were gated for live (FSC-A and SSC-A) and single cell (FSC-A and FSC-H) and percentage of GFP+ cells was quantified (right). Top row: noE. Middle row: E0. Bottom row: E0, 31 days after sort. GFP-gating is based on noE, showing that ~2% of the cells became GFP-negative.
