# Supplementary material for: Building regulatory landscapes reveals that an enhancer can recruit cohesin to create contact domains, engage CTCF sites and activate distant genes
Source: Nat Struct Mol Biol. 2022 Jun 16;29(6):563–74. doi: 10.1038/s41594-022-00787-7 (PMC9205769; doi:10.1038/s41594-022-00787-7)
Supplement: Supplementary file 2 — Reporting Summary. [file 41594_2022_787_MOESM2_ESM.pdf]

nature portfolio

Corresponding author(s): Wouter de Laat

Last updated by author(s): Mar 21st 2022

Reporting Summary

Nature Portfolio wishes to improve the reproducibility of the work that we publish. This form provides structure for consistency and transparency in reporting. For further information on Nature Portfolio policies, see our Editorial Policies and the Editorial Policy Checklist.

Statistics

For all statistical analyses, confirm that the following items are present in the figure legend, table legend, main text, or Methods section.

n/a	Confirmed	
☐	☒	The exact sample size (*n*) for each experimental group/condition, given as a discrete number and unit of measurement
☐	☒	A statement on whether measurements were taken from distinct samples or whether the same sample was measured repeatedly
☒	☐	The statistical test(s) used AND whether they are one- or two-sided<br>*Only common tests should be described solely by name; describe more complex techniques in the Methods section.*
☒	☐	A description of all covariates tested
☒	☐	A description of any assumptions or corrections, such as tests of normality and adjustment for multiple comparisons
☐	☒	A full description of the statistical parameters including central tendency (e.g. means) or other basic estimates (e.g. regression coefficient) AND variation (e.g. standard deviation) or associated estimates of uncertainty (e.g. confidence intervals)
☒	☐	For null hypothesis testing, the test statistic (e.g. *F*, *t*, *r*) with confidence intervals, effect sizes, degrees of freedom and *P* value noted<br>*Give P values as exact values whenever suitable.*
☒	☐	For Bayesian analysis, information on the choice of priors and Markov chain Monte Carlo settings
☒	☐	For hierarchical and complex designs, identification of the appropriate level for tests and full reporting of outcomes
☒	☐	Estimates of effect sizes (e.g. Cohen's *d*, Pearson's *r*), indicating how they were calculated

Our web collection on statistics for biologists contains articles on many of the points above.

Software and code

Policy information about availability of computer code

Data collection	CytExpert 2.3.0.84, Bio-Rad CFX Manager 2.1
Data analysis	Plots were generated in R (version 4.1.0), FlowJo 10.5.2 and GraphPad Prism 8.2.1. Sequencing was analyzed with pipeline "pipe4C" (https://github.com/deLaatLab/pipe4C) and custom R base code, as indicated in text.

For manuscripts utilizing custom algorithms or software that are central to the research but not yet described in published literature, software must be made available to editors and reviewers. We strongly encourage code deposition in a community repository (e.g. GitHub). See the Nature Portfolio guidelines for submitting code & software for further information.

Data

Policy information about availability of data

All manuscripts must include a data availability statement. This statement should provide the following information, where applicable:

- Accession codes, unique identifiers, or web links for publicly available datasets
- A description of any restrictions on data availability
- For clinical datasets or third party data, please ensure that the statement adheres to our policy

Public ChIP-seq and RNA-seq (mapped to human genome release 19) tracks were downloaded from ENCODE portal9. The following data-sets from ENCODE were used: K562 SMC3 ChIP-seq (Encode, Michael Snyder, ENCSR000EGW, ENCFF479BWQ), ChIP-seq CTCF ChIP-seq (Encode, Bradley Bernstein, ENCSR000AKO, ENCFF000BWF), K562 RNA-seq (Encode, Brenton Graveley, ENCSR000AEN, ENCFF657EOD, ENCFF578WIM). H3K27ac (Encode, Bradley Bernstein, GEO: GSM733656) and H3K27me3 (Encode, Michael Snyder, GEO: GSM788088).

Raw sequencing data and mapped wig files of the following experiments are available without restriction from the Gene Expression Omnibus (GEO) under accession

GSE180566:

Sample Technique Genotype Viewpoint Hemin Sorted KnockDown Genome Figure
Sample_1 4C WT Pos0 no_Hemin GFP_negative NA hg19_with_insert 2a
Sample_2 4C WT Pos0 no_Hemin GFP_negative NA hg19_with_insert 2a
Sample_3 4C noE Pos0 no_Hemin GFP_negative NA hg19_with_insert 2c, ext. 3
Sample_4 4C noE Pos0 no_Hemin GFP_negative NA hg19_with_insert 2c, ext. 3
Sample_5 4C noE GFP no_Hemin GFP_negative NA hg19_with_insert 2a, 2b, 2c, 2e, 4b, 4c, 4j, ext. 2c
Sample_6 4C noE GFP no_Hemin GFP_negative NA hg19_with_insert 2a, 2b, 2c, 2e, 4b, 4c, 4j, ext. 2c
Sample_7 4C noE GFP no_Hemin GFP_negative NA hg19_with_insert 2a, 2b, 2c, 2e, 4b, 4c, 4j, ext. 2c
Sample_8 4C E0 GFP no_Hemin 2xGFP_positive NA hg19_with_insert 1j, 2b, 2c, 3c, ext. 2a
Sample_9 4C E0 GFP no_Hemin 2xGFP_positive NA hg19_with_insert 1j, 2b, 2c, 3c, ext. 2a
Sample_10 4C E11 GFP no_Hemin 2xGFP_positive NA hg19_with_insert 1j, 2b, 2c, ext. 2a
Sample_11 4C E11 GFP no_Hemin 2xGFP_positive NA hg19_with_insert 1j, 2b, 2c, ext. 2a
Sample_12 4C E47 GFP no_Hemin 2xGFP_positive NA hg19_with_insert 1j, 2b, 2c, ext. 2a
Sample_13 4C E47 GFP no_Hemin 2xGFP_positive NA hg19_with_insert 1j, 2b, 2c, ext. 2a
Sample_14 4C E100 GFP no_Hemin 2xGFP_positive NA hg19_with_insert 1j, 2b, 2c, 3c, 3e, 4b, ext. 2a
Sample_15 4C E100 GFP no_Hemin 2xGFP_positive NA hg19_with_insert 1j, 2b, 2c, 3c, 3e, 4b, ext. 2a
Sample_16 4C E407 GFP no_Hemin 2xGFP_positive NA hg19_with_insert 1j, 2e, 2d, ext. 2a
Sample_17 4C E407 GFP no_Hemin 2xGFP_positive NA hg19_with_insert 1j, 2e, 2d, ext. 2a
Sample_18 4C E407 GFP no_Hemin 2xGFP_positive NA hg19_with_insert 1j, 2e, 2d, ext. 2a
Sample_19 4C EC0 GFP no_Hemin 2xGFP_positive NA hg19_with_insert 3c, 3d
Sample_20 4C EC0 GFP no_Hemin 2xGFP_positive NA hg19_with_insert 3c, 3d
Sample_21 4C EC0 GFP no_Hemin 2xGFP_positive NA hg19_with_insert 3c, 3d
Sample_22 4C E0 uLCR no_Hemin 2xGFP_positive NA hg19_with_insert 2c, 3c, ext. 3
Sample_23 4C E0 uLCR no_Hemin 2xGFP_positive NA hg19_with_insert 2c, 3c, ext. 3
Sample_24 4C EC0 uLCR no_Hemin 2xGFP_positive NA hg19_with_insert 3c
Sample_25 4C EC0 uLCR no_Hemin 2xGFP_positive NA hg19_with_insert 3c
Sample_26 4C EC0 uLCR no_Hemin 2xGFP_positive NA hg19_with_insert 3c
Sample_27 4C EC100 GFP no_Hemin 2xGFP_positive NA hg19_with_insert 3c, 3d, 4c
Sample_28 4C EC100 GFP no_Hemin 2xGFP_positive NA hg19_with_insert 3c, 3d, 4c
Sample_29 4C E100 uLCR no_Hemin 2xGFP_positive NA hg19_with_insert 2c, 3c, 3e, 4b, ext. 3
Sample_30 4C E100 uLCR no_Hemin 2xGFP_positive NA hg19_with_insert 2c, 3c, 3e, 4b, ext. 3
Sample_31 4C EC100 uLCR no_Hemin 2xGFP_positive NA hg19_with_insert 3c, 4c
Sample_32 4C EC100 uLCR no_Hemin 2xGFP_positive NA hg19_with_insert 3c, 4c
Sample_33 4C EC100 uLCR no_Hemin 2xGFP_positive NA hg19_with_insert 3c, 4c
Sample_34 4C C0 GFP no_Hemin GFP_negative NA hg19_with_insert 3d
Sample_35 4C C0 GFP no_Hemin GFP_negative NA hg19_with_insert 3d
Sample_36 4C C0 GFP no_Hemin GFP_negative NA hg19_with_insert 3d
Sample_37 4C C0 CBS no_Hemin GFP_negative NA hg19_with_insert 3d
Sample_38 4C C0 CBS no_Hemin GFP_negative NA hg19_with_insert 3d
Sample_39 4C EC0 CBS no_Hemin 2xGFP_positive NA hg19_with_insert 3d
Sample_40 4C EC0 CBS no_Hemin 2xGFP_positive NA hg19_with_insert 3d
Sample_41 4C C100 GFP no_Hemin GFP_negative NA hg19_with_insert 3d
Sample_42 4C C100 GFP no_Hemin GFP_negative NA hg19_with_insert 3d
Sample_43 4C C100 CBS no_Hemin GFP_negative NA hg19_with_insert 3d
Sample_44 4C C100 CBS no_Hemin GFP_negative NA hg19_with_insert 3d
Sample_45 4C EC100 CBS no_Hemin 2xGFP_positive NA hg19_with_insert 3d, 4c
Sample_46 4C EC100 CBS no_Hemin 2xGFP_positive NA hg19_with_insert 3d, 4c
Sample_47 4C C-0E100 GFP no_Hemin 2xGFP_positive NA hg19_with_insert 3e
Sample_48 4C C-0E100 GFP no_Hemin 2xGFP_positive NA hg19_with_insert 3e
Sample_49 4C C-0E100 GFP no_Hemin 2xGFP_positive NA hg19_with_insert 3e
Sample_50 4C C-0E100 uLCR no_Hemin 2xGFP_positive NA hg19_with_insert 3e
Sample_51 4C C-0E100 uLCR no_Hemin 2xGFP_positive NA hg19_with_insert 3e
Sample_52 4C C-0E100 uLCR no_Hemin 2xGFP_positive NA hg19_with_insert 3e
Sample_53 4C E100 GFP no_Hemin 2xGFP_positive Control_KD hg19_with_insert 4a
Sample_54 4C E100 GFP no_Hemin 2xGFP_positive SMC1A_KD hg19_with_insert 4a, 4b
Sample_55 4C EC100 GFP no_Hemin 2xGFP_positive Control_KD hg19_with_insert 4a
Sample_56 4C EC100 GFP no_Hemin 2xGFP_positive Control_KD hg19_with_insert 4a
Sample_57 4C EC100 GFP no_Hemin 2xGFP_positive SMC1A_KD hg19_with_insert 4a, 4c
Sample_58 4C E100 GFP no_Hemin 2xGFP_positive RAD21_KD hg19_with_insert 4b, ext. 5
Sample_59 4C noE Pos100 no_Hemin GFP_negative NA hg19_with_insert 2c, 4b, 4c, ext. 3
Sample_60 4C noE Pos100 no_Hemin GFP_negative NA hg19_with_insert 2c, 4b, 4c, ext. 3
Sample_61 4C E100 uLCR no_Hemin 2xGFP_positive SMC1A_KD hg19_with_insert 4b, ext. 5
Sample_62 4C E100 uLCR no_Hemin 2xGFP_positive RAD21_KD hg19_with_insert 4b, ext. 5
Sample_63 4C EC100 GFP no_Hemin 2xGFP_positive RAD21_KD hg19_with_insert 4c, ext. 5
Sample_64 4C EC100 uLCR no_Hemin 2xGFP_positive SMC1A_KD hg19_with_insert 4c, ext. 5
Sample_65 4C EC100 uLCR no_Hemin 2xGFP_positive RAD21_KD hg19_with_insert 4c, ext. 5
Sample_66 4C EC100 CBS no_Hemin 2xGFP_positive SMC1A_KD hg19_with_insert 4c, ext. 5
Sample_67 4C EC100 CBS no_Hemin 2xGFP_positive RAD21_KD hg19_with_insert 4c, ext. 5
Sample_68 4C E407 GFP no_Hemin 2xGFP_positive Control_KD_ hg19_with_insert 4d, ext. 5
Sample_69 4C E407 GFP no_Hemin 2xGFP_positive Control_KD_ hg19_with_insert 4d, ext. 5
Sample_70 4C E407 GFP no_Hemin 2xGFP_positive Control_KD_ hg19_with_insert 4d, ext. 5
Sample_71 4C E407 GFP no_Hemin 2xGFP_positive Control_SMC1A_ hg19_with_insert 4d
Sample_72 4C E407 uLCR no_Hemin 2xGFP_positive Control_KD_ hg19_with_insert 4d, ext. 5
Sample_73 4C E407 uLCR no_Hemin 2xGFP_positive Control_KD_ hg19_with_insert 4d, ext. 5

Sample_74 4C E407 uLCR no_Hemin 2xGFP_positive SMC1A_KD_ hg19_with_insert 4d
Sample_75 4C E0C0C100 GFP no_Hemin 2xGFP_positive NA hg19_with_insert 4j
Sample_76 4C E0C0C100 GFP no_Hemin 2xGFP_positive NA hg19_with_insert 4j
Sample_77 4C E100C0C100 GFP no_Hemin 2xGFP_positive NA hg19_with_insert 4j
Sample_78 4C E100C0C100 GFP no_Hemin 2xGFP_positive NA hg19_with_insert 4j
Sample_79 4C E0 GFP plus_Hemin 2xGFP_positive NA hg19_with_insert 1j, ext. 2a
Sample_80 4C E0 GFP plus_Hemin 2xGFP_positive NA hg19_with_insert 1j, ext. 2a
Sample_81 4C E11 GFP plus_Hemin 2xGFP_positive NA hg19_with_insert 1j, ext. 2a
Sample_82 4C E11 GFP plus_Hemin 2xGFP_positive NA hg19_with_insert 1j, ext. 2a
Sample_83 4C E47 GFP plus_Hemin 2xGFP_positive NA hg19_with_insert 1j, ext. 2a
Sample_84 4C E47 GFP plus_Hemin 2xGFP_positive NA hg19_with_insert 1j, ext. 2a
Sample_85 4C E100 GFP plus_Hemin 2xGFP_positive NA hg19_with_insert 1j, ext. 2a
Sample_86 4C E100 GFP plus_Hemin 2xGFP_positive NA hg19_with_insert 1j, ext. 2a
Sample_87 4C E100 GFP plus_Hemin 2xGFP_positive NA hg19_with_insert 1j, ext. 2a
Sample_88 4C E407 GFP plus_Hemin 2xGFP_positive NA hg19_with_insert 1j, ext. 2a
Sample_89 4C E407 GFP plus_Hemin 2xGFP_positive NA hg19_with_insert 1j, ext. 2a
Sample_90 4C E407 GFP plus_Hemin 2xGFP_positive NA hg19_with_insert 1j, ext. 2a
Sample_91 4C E100 GFP no_Hemin 6xGFP_negative NA hg19_with_insert 1j
Sample_92 4C E100 GFP no_Hemin 6xGFP_negative NA hg19_with_insert 1j
Sample_96 4C E407 GFP no_Hemin 9xGFP_negative NA hg19_with_insert 1j
Sample_97 4C E407 GFP no_Hemin 9xGFP_negative NA hg19_with_insert 1j
Sample_98 4C E407 GFP no_Hemin 9xGFP_negative NA hg19_with_insert 1j
Sample_101 4C noE Pos11 no_Hemin GFP_negative NA hg19_with_insert 2c, ext. 3
Sample_102 4C noE Pos11 no_Hemin GFP_negative NA hg19_with_insert 2c, ext. 3
Sample_105 4C noE Pos47 no_Hemin GFP_negative NA hg19_with_insert 2c, ext. 3
Sample_106 4C noE Pos47 no_Hemin GFP_negative NA hg19_with_insert 2c, ext. 3
Sample_111 4C noE Pos407 no_Hemin GFP_negative NA hg19_with_insert 2d, ext. 3
Sample_112 4C noE Pos407 no_Hemin GFP_negative NA hg19_with_insert 2d, ext. 3
Sample_113 4C E11 uLCR no_Hemin 2xGFP_positive NA hg19_with_insert 2c, ext. 3
Sample_114 4C E11 uLCR no_Hemin 2xGFP_positive NA hg19_with_insert 2c, ext. 3
Sample_115 4C E47 uLCR no_Hemin 2xGFP_positive NA hg19_with_insert 2c, ext. 3
Sample_116 4C E47 uLCR no_Hemin 2xGFP_positive NA hg19_with_insert 2c, ext. 3
Sample_117 4C E407 uLCR no_Hemin 2xGFP_positive NA hg19_with_insert 2d, ext. 3
Sample_118 4C E407 uLCR no_Hemin 2xGFP_positive NA hg19_with_insert 2d, ext. 3
Sample_119 4C E407 uLCR no_Hemin 2xGFP_positive NA hg19_with_insert 2d, ext. 3
Sample_120 4C noE LeftBoundary no_Hemin GFP_negative NA hg19_with_insert ext. 4
Sample_121 4C noE LeftBoundary no_Hemin GFP_negative NA hg19_with_insert ext. 4
Sample_122 4C EC0 LeftBoundary no_Hemin 2xGFP_positive NA hg19_with_insert ext. 4
Sample_123 4C EC0 LeftBoundary no_Hemin 2xGFP_positive NA hg19_with_insert ext. 4
Sample_124 4C EC100 LeftBoundary no_Hemin 2xGFP_positive NA hg19_with_insert ext. 4
Sample_125 4C EC100 LeftBoundary no_Hemin 2xGFP_positive NA hg19_with_insert ext. 4
Sample_126 4C E0C0C100 LeftBoundary no_Hemin 2xGFP_positive NA hg19_with_insert ext. 4
Sample_127 4C E0C0C100 LeftBoundary no_Hemin 2xGFP_positive NA hg19_with_insert ext. 4
Sample_128 4C E100C0C100 LeftBoundary no_Hemin 2xGFP_positive NA hg19_with_insert ext. 4
Sample_129 4C E100C0C100 LeftBoundary no_Hemin 2xGFP_positive NA hg19_with_insert ext. 4
Sample_130 4C EC100 uLCR no_Hemin 2xGFP_positive Control_KD hg19_with_insert ext. 5
Sample_131 4C EC100 GFP no_Hemin 2xGFP_positive Control_KD hg19_with_insert ext. 5
Sample_132 4C EC100 GFP no_Hemin 2xGFP_positive Control_KD hg19_with_insert ext. 5
Sample_133 4C EC100 uLCR no_Hemin 2xGFP_positive Control_KD hg19_with_insert ext. 5
Sample_134 4C EC100 uLCR no_Hemin 2xGFP_positive Control_KD hg19_with_insert ext. 5
Sample_135 4C EC100 CBS no_Hemin 2xGFP_positive Control_KD hg19_with_insert ext. 5
Sample_136 4C EC100 CBS no_Hemin 2xGFP_positive Control_KD hg19_with_insert ext. 5
Sample_137 4C E407 GFP no_Hemin 2xGFP_positive RAD21_KD hg19_with_insert ext. 5
Sample_138 4C E407 uLCR no_Hemin 2xGFP_positive RAD21_KD hg19_with_insert ext. 5
Sample_139 4C EC100 CBS no_Hemin 2xGFP_positive Control_KD hg19_with_insert ext. 5
Sample_140 4C EC100 CBS no_Hemin 2xGFP_positive Control_KD hg19_with_insert ext. 5
Sample_141 4C E47 GFP no_Hemin 2xGFP_positive NA hg19_with_insert 1j
Sample_142 4C E47 GFP no_Hemin 2xGFP_positive NA hg19_with_insert 1j
Sample_143 4C E100 GFP no_Hemin 2xGFP_positive NA hg19_with_insert 1j, ext. 2b
Sample_144 4C E100 GFP no_Hemin 2xGFP_positive NA hg19_with_insert 1j, ext. 2b
Sample_145 4C E407 GFP no_Hemin 2xGFP_positive NA hg19_with_insert 1j, ext. 2b
Sample_146 4C E407 GFP no_Hemin 2xGFP_positive NA hg19_with_insert 1j, ext. 2b
Sample_147 4C E100 GFP no_Hemin 6xGFP_negative NA hg19_with_insert 1j, ext. 2b, 2c
Sample_148 4C E100 GFP no_Hemin 6xGFP_negative NA hg19_with_insert 1j, ext. 2b, 2c
Sample_149 4C E407 GFP no_Hemin 9xGFP_negative NA hg19_with_insert 1j, ext. 2b, 2c
Sample_150 4C E407 GFP no_Hemin 9xGFP_negative NA hg19_with_insert 1j, ext. 2b, 2c
Sample_151 4C E407 GFP no_Hemin 9xGFP_negative NA hg19_with_insert 1j
Sample_152 4C E407 GFP no_Hemin 9xGFP_negative NA hg19_with_insert 1j
Sample_153 4C C0 LeftBoundary no_Hemin GFP_negative NA hg19_with_insert ext. 4
Sample_154 4C C0 LeftBoundary no_Hemin GFP_negative NA hg19_with_insert ext. 4
Sample_155 4C C0 LeftBoundary no_Hemin GFP_negative NA hg19_with_insert ext. 4
Sample_156 4C C100 LeftBoundary no_Hemin GFP_negative NA hg19_with_insert ext. 4
Sample_157 4C C100 LeftBoundary no_Hemin GFP_negative NA hg19_with_insert ext. 4

Field-specific reporting

Please select the one below that is the best fit for your research. If you are not sure, read the appropriate sections before making your selection.

☒ Life sciences ☐ Behavioural & social sciences ☐ Ecological, evolutionary & environmental sciences

For a reference copy of the document with all sections, see nature.com/documents/nr-reporting-summary-flat.pdf

Life sciences study design

All studies must disclose on these points even when the disclosure is negative.

Sample size	No statistical method was used to predetermine sample size
Data exclusions	no data were excluded from the analyses
Replication	Number of replicates are indicated in the figure legends. All 4C and all but one ChIP-qPCR experiments represent at least two technical replicates.<br>Additionally, all conclusions are based on the analysis of multiple independent clones having the enhancer at different distances and/or having ectopic CTCF sites at different positions and/or having different cohesin subunits depleted.
Randomization	the experiments were not randomized
Blinding	The investigators were not blinded to allocation during experiments and outcome assessment.

Reporting for specific materials, systems and methods

We require information from authors about some types of materials, experimental systems and methods used in many studies. Here, indicate whether each material, system or method listed is relevant to your study. If you are not sure if a list item applies to your research, read the appropriate section before selecting a response.

Materials & experimental systems

n/a	Involved in the study	
☐	☒	Antibodies
☐	☒	Eukaryotic cell lines
☒	☐	Palaeontology and archaeology
☒	☐	Animals and other organisms
☒	☐	Human research participants
☒	☐	Clinical data
☒	☐	Dual use research of concern

Methods

n/a	Involved in the study	
☒	☐	ChIP-seq
☐	☒	Flow cytometry
☒	☐	MRI-based neuroimaging

Antibodies

Antibodies used	SMC1: A300-055A, for western 1:1000, for ChIP 5ug per 30ug of chromatin, Bethyl, https://www.thermofisher.com/antibody/product/SMC1-Antibody-Polyclonal/A300-055A<br>CTCF: 07-829, ChIP 5ug per 30ug of chromatin, Millipore, https://www.merckmillipore.com/NL/en/product/Anti-CTCF-Antibody,MM_NF-07-729?ReferrerURL=https%3A%2F%2Fwww.google.com%2F<br>H3K27me3: ab6002, ChIP 5ug per 30ug of chromatin, Abcam, https://www.abcam.com/Histone-H3-tri-methyl-K27-antibody-mAbcam-6002-ChIP-Grade-ab6002.html?gclsrc=aw.ds|aw.ds&gclid=CjwKCAjwxOCRBhA8EiwA0X8hi4XuFW8mffpMVkwU6GjRPPeanemAtd_AXf5jc85V5YQ2EJR1ftOxJhoCL9oQAvD_BwE<br>γ-Tubulin: GTU-88 T6557, 1:3000, Sigma, https://www.sigmaaldrich.com/NL/en/product/sigma/t6557<br>goat anti rabbit HRP: #7074, 1:3000, Cell Signalling, https://www.cellsignal.com/products/secondary-antibodies/anti-rabbit-igg-hrp-linked-antibody/7074<br>goat anti mouse HRP: #7076, 1:3000, Cell, Signalling, https://www.cellsignal.com/products/secondary-antibodies/anti-mouse-igg-hrp-linked-antibody/7076
Validation	Antibody validation can be found on manufacturers website as indicated.

Eukaryotic cell lines

Policy information about cell lines

Cell line source(s)	Human erythroleukemia K562 cells were used in this study (not authenticated cell line, available in our institute, periodically tested for mycoplasma).
Authentication	K562 cells were not authenticated.
Mycoplasma contamination	K562 cells were regularly testes for mycoplasma.
Commonly misidentified lines (See ICLAC register)	No commonly misidentified cell lines were used.

Flow Cytometry

Plots

Confirm that:

☒ The axis labels state the marker and fluorochrome used (e.g. CD4-FITC).

☒ The axis scales are clearly visible. Include numbers along axes only for bottom left plot of group (a 'group' is an analysis of identical markers).

☒ All plots are contour plots with outliers or pseudocolor plots.

☒ A numerical value for number of cells or percentage (with statistics) is provided.

Methodology

Sample preparation	For flow cytometry analysis, genetically modified K562 cells were cultured as indicated in methods section and either treated with or without hemin two days prior to analysis. Cells were transferred to a 96-wells plate for flow cytometry analysis directly from the culture dish. For every experiment, at least 10,000 cells within the live single-cell gate were recorded.<br>For fluorescence assisted cell sorting, 10 million cells were collected, centrifuged and resuspended in 1 mL of culture medium.
Instrument	Flow cytometry analysis: Cytoflex S, Beckman Coulter Inc. Model No.: B75442<br>Fluorescence assisted cell sorting: FACS Aria Fusion, Beckman Coulter Inc. Special Order Research Product
Software	Flow cytometry analysis: CytExpert 2.3.0.84<br>Fluorescence assisted cell sorting: FACSDiva 8.0.1, Beckman Coulter Inc.
Cell population abundance	We used identical gate settings to separate GFP (or dsRed) positive and negative cells for all experiments, as described in the manuscript.
Gating strategy	For flow cytometry analysis and fluorescence assisted cell sorting, cells were gated for live single cells based on FSC-A, SSC-A and FSC-H.<br>For sorting, cells were considered GFP positive based on gating on "noE" cell line. Gates were drawn such that more than 99.9% of "noE" cells felt into the GFP-negative gate.<br>To enrich for KRAB::BFP containing cells prior to knock-down experiments, cells were gated for BFP positive. From this population, the top 50% BFP expressing cells were sorted.<br>For analysis of knock-down experiments, cells were gated to be BFP/sgRNA positive as compared to cells that only contained KRAB::BFP.

☒ Tick this box to confirm that a figure exemplifying the gating strategy is provided in the Supplementary Information.
