# Supplementary material for: Building regulatory landscapes reveals that an enhancer can recruit cohesin to create contact domains, engage CTCF sites and activate distant genes
Source: Nat Struct Mol Biol. 2022 Jun 16;29(6):563–74. doi: 10.1038/s41594-022-00787-7 (PMC9205769; doi:10.1038/s41594-022-00787-7)
Supplement: Source Data Fig. 1 — Unprocessed Western Blots from main figure 5c [file 41594_2022_787_MOESM4_ESM.pdf]

SMC1A antibody (rabbit, Bethyl A300-055A, 1:1000)

Blot 1: E0-E100

Blot 2: E407

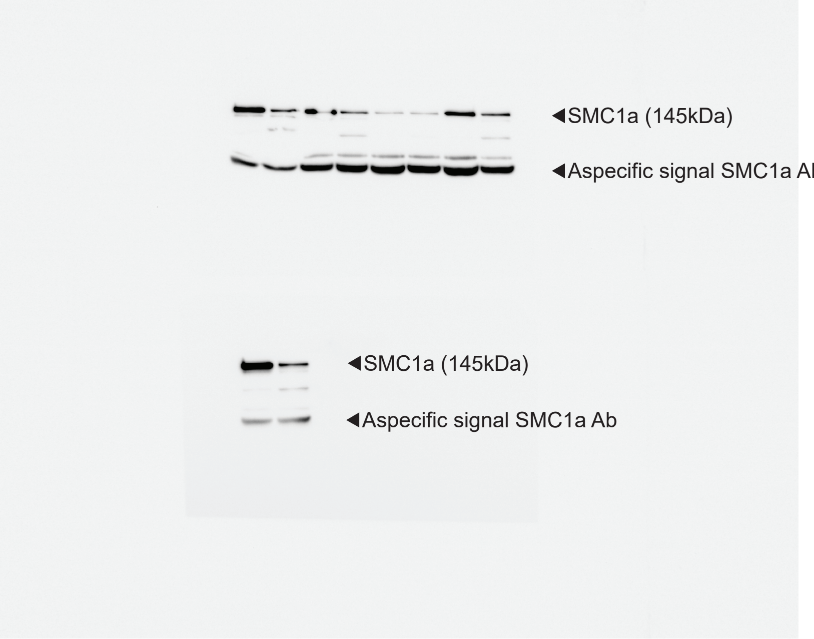

gTubulin (GTU-88) antibody (mouse, Sigma #T6557, 1:1000)

Blot 1: E0-E100 (stripped)

Blot 2: E407 (stripped)

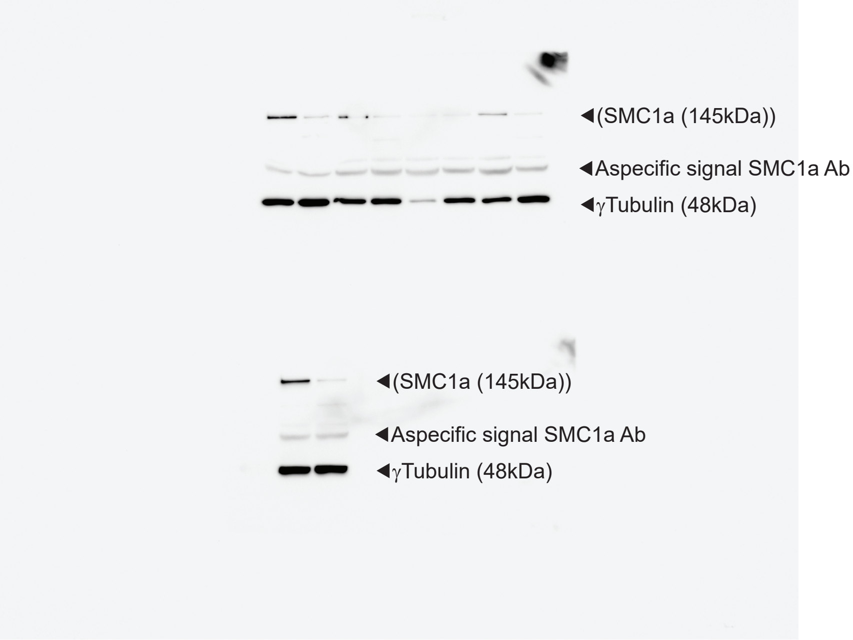

Source Data Fig. 1 | Unprocessed SMC1A immunoblot scan

Unprocessed scans corresponding to main figure 5c. Upper scan are two blots stained for SMC1A, lower scan shows same blots, stripped and stained for γTubulin. SMC1A and γTubulin bands are indicated. Gel1 (top) contains from left to right: E0 control KD (C), E0 SMC1A knockdown (KD), E11 C, E11 KO, E47 C, E47 KO, E100 C, E100 KO. Gel2 (bottom) contains E407 C and E407 KO.
